# Editorial: Targeting dysregulation in metabolic diseases: potential therapeutic intervention

**DOI:** 10.3389/fphar.2026.1995384

**Published:** 2026-09-25

**Authors:** Vítor S. Fernandes, Yoshinori Moriyama

**Affiliations:** 1 Department of Physiology, Faculty of Pharmacy, Complutense University of Madrid, Madrid, Spain; 2 Department of Internal Medicine, Kurume University School of Medicine, Kurume, Fukuoka, Japan

**Keywords:** immunometabolism, inflammation, metabolic diseases, metabolic dysregulation, precision medicine, therapeutic targets

## Introduction

Metabolic diseases comprise a broad and heterogeneous group of disorders characterized by dysregulation of nutrient and energy homeostasis, endocrine and intracellular signaling, organelle function, redox balance, inflammatory pathways, and inter-organ metabolic communication. Diabetes, obesity, and related conditions therefore cannot be fully characterized by abnormalities in individual metabolic biomarkers alone. Their pathophysiology involves interconnected molecular, cellular, tissue, and systemic mechanisms that contribute to multisystem complications and affect both therapeutic target identification and clinical management.

The Research Topic “*Targeting Dysregulation in Metabolic Diseases: Potential Therapeutic Intervention*” brings together 23 contributions, including reviews, systematic reviews and meta-analyses, original preclinical investigations, and clinical studies. Collectively, these articles address diabetes and its complications, metabolic dysfunction-associated steatotic liver disease (MASLD), metabolic dysfunction-associated steatohepatitis (MASH), osteoporosis, atherosclerosis, hyperuricemia, reproductive dysfunction, metabolic alterations in high-risk pregnancies, and metabolic syndrome. They explore pharmacological and non-pharmacological interventions ranging from targeted and repurposed drugs to natural products, botanical formulations, nutritional strategies, probiotics, and physical activity. Across these topics, several contributions incorporate transcriptomics, proteomics, metabolomics, systems biology, network pharmacology, molecular modeling, and machine-learning approaches to define disease mechanisms, candidate biomarkers, and therapeutic targets. Together, they illustrate how mechanistic insight, target identification, and progressive experimental and clinical validation can be integrated to support more precise and effective therapeutic approaches to metabolic disease.

This Editorial is organized into three complementary sections that mirror the collection’s scientific content. “*Metabolic Dysregulation as a Networked Process*” explores the molecular determinants of metabolic dysfunction and identifies regulatory nodes and potential therapeutic targets. “*Cellular Homeostasis, Organ-Specific Disease, and Inter-Organ Communication*” addresses organelle homeostasis, organ-specific pathophysiology, tissue remodeling, and systemic/inter-organ regulation across diabetic complications, liver disease, osteoporosis, reproductive dysfunction, high-risk pregnancy, and hyperuricemia. Finally, “*Therapeutic Evidence and Clinical Translation*” evaluates the strength and translational maturity of therapeutic evidence, from preclinical and mechanistic studies to randomized trials, systematic reviews, meta-analyses, and observational clinical research. Taken together, this Research Topic emphasizes that a better understanding of the molecular, cellular, organ-specific, and systemic mechanisms underlying metabolic diseases is essential to developing more effective and targeted therapeutic strategies.

## Metabolic dysregulation as a networked process

Several studies in this Research Topic show that metabolic dysregulation involves more than just glucose and lipid levels. For instance, Hasuzawa et al. focus on extracellular ATP released through vesicular exocytosis and identify the vesicular nucleotide transporter VNUT/SLC17A9 as a regulatory point linking cellular bioenergetics with purinergic signaling, inflammation, sensory transmission, metabolic dysregulation, and other pathological processes. This places controlled ATP exocytosis upstream of multiple extracellular responses and suggests that inhibiting VNUT-dependent release may modulate disease networks rather than a single downstream mediator. In parallel, Chen et al. review phenylalanine homeostasis and show that phenylalanine is not merely an amino-acid substrate. Epidemiological and mechanistic evidence links phenylalanine dysregulation with obesity, type 2 diabetes and other metabolic disorders, while phenylalanylation of the insulin-receptor beta subunit can impair insulin signaling; the reversibility of this modification through SIRT1 further illustrates how metabolite handling and post-translational regulation intersect.

A similar shift from quantity to molecular quality appears in Shimizu et al., who emphasize that hepatic triglyceride burden alone cannot explain insulin resistance and metabolic dysfunction in type 2 diabetes. Fatty-acid composition and phospholipid diversity, including chain length, unsaturation, and omega-3/omega-6 characteristics, also shape hepatic metabolic regulation. Fang et al. extend this context dependence to endocrine modulators in MASLD. Their synthesis distinguishes the relatively protective actions of IGFBP1 and IGFBP2, the stage-dependent behavior of IGFBP3 and IGFBP5, a predominantly pathogenic role for IGFBP7, and the less-defined contributions of IGFBP4 and IGFBP6. This has important implications for target development because the same molecular family can exert protective, maladaptive, or stage-specific effects, making disease stage and biological compartment essential components of pharmacological interpretation.

Two original studies illustrate how these network concepts can be converted into target-discovery pipelines. Naeem et al. investigated the antiemetic ondansetron and newly synthesized derivatives as inhibitors of protein tyrosine phosphatase 1B (PTP1B), a negative regulator of insulin-receptor signaling. Their combined *in silico*, *in vitro*, and *in vivo* workflow identified FN-06 and FN-10 as lead structures with PTP1B-related activity and antidiabetic and hepatoprotective effects in animal experiments, supporting drug repurposing and derivative optimization while remaining at a preclinical stage. Wang et al. integrated transcriptomics, weighted gene co-expression analysis, machine learning, virtual screening, molecular dynamics, and experimental validation to prioritize SIRT1 as a senescence-related candidate in atherosclerosis. Poliumoside emerged from a 5,565-compound natural-product screen, and its protection against oxidative endothelial injury involved the SIRT1/NRF2/HO-1 axis and was attenuated by SIRT1 knockdown. These studies exemplify a transition from phenotype-first screening to network-informed identification and validation of regulatory nodes.

## Cellular homeostasis, organ-specific disease, and inter-organ communication

Organelle quality control is another recurring layer of dysregulation. Chen et al. review lysosomes as degradative, secretory, and signaling organelles whose dysfunction can disturb autophagy, nutrient recycling, cellular signaling, and energy homeostasis in diabetes and its chronic complications. Zhao et al. provide a complementary systems-level perspective on diabetic retinopathy using a paired longitudinal design. In 15 individuals who progressed from diabetes without retinopathy to diabetic retinopathy over 1–2 years, plasma proteomic and metabolomic profiling identified progression-associated molecular changes; eight proteins were subsequently evaluated by ELISA in an independent cohort, including established retinopathy-associated proteins and five proposed candidates (ALDH18A1, GALT, PRG2, UBA1, and GCG). This work illustrates both the promise and the need for external validation of circulating multi-omic signatures for early risk stratification.

Diabetic peripheral neuropathy further demonstrates why mechanistic plausibility must be connected to pharmacokinetics. Liu et al. critically assess paeoniflorin as a potential disease-modifying agent acting across oxidative stress (Nrf2/HO-1), neuroinflammation (TLR4/NF-kappaB), neurotrophic repair (BDNF/TrkB), and microvascular pathways (including HIF-1alpha/VEGF). At the same time, they emphasize very low oral bioavailability, presystemic biotransformation, uncertainty regarding the active molecular species, and insufficient measurements of drug exposure in peripheral nerve and dorsal-root ganglia. Thus, a multitarget mechanism is therapeutically meaningful only if adequate target-tissue exposure can be demonstrated. In MASH, Wang et al. combined experiments in HepG2 cells with an HFD/CCl_4_ mouse model, transcriptomic and metabolomic analyses, and molecular validation. Artemisia scoparia extract improved lipid and inflammatory abnormalities, modulated dysregulated genes and metabolites, and attenuated oxidative stress and ferroptosis, while modulating AGE–RAGE and JAK1–STAT3 signaling. Jiménez-González et al. address MASLD from a repurposing perspective, suggesting the mevalonate pathway and reduced prenylation of small GTPases as a shared substrate through which statins may influence lipotoxicity, NLRP3-related inflammation, fibrogenesis, carcinogenesis, and intrahepatic hemodynamics beyond LDL lowering. Importantly, they distinguish predominantly observational and indirect evidence from randomized evidence and identify decompensated cirrhosis as a stage in which clinical benefit has not been demonstrated, reinforcing the concept of a therapeutic window.

Osteoporosis is represented by four contributions that collectively move from intracellular bioenergetics to multicellular remodeling and systemic regulation. One review by Li and Liu frames mitochondrial biogenesis, dynamics, oxidative stress, and mitophagy as targets through which Chinese botanical drugs may influence osteoblast-osteoclast homeostasis. Another review presented by the same authors, Li and Liu, focuses on the gut-bone axis, integrating microbiota composition, microbial metabolites, intestinal barrier function, endocrine signaling, and immune regulation. Uehara et al. refine the cellular target further by considering osteoclast precursor fusion and functional polarization. Strategies aimed at DC-STAMP/OC-STAMP, Siglec-15, Dock5, LRRK1, and related mechanisms could, in principle, reduce pathological resorption while preserving osteoclast-osteoblast coupling more selectively than global antiresorptive suppression. Finally, Wang et al. synthesize evidence for plants used in medicines and foods, describing coordinated effects on Wnt/beta-catenin and BMP/SMAD osteogenesis, RANKL/NFATc1/NF-kappaB osteoclastogenesis, endocrine pathways, the gut-bone/immune environment, and age-related bone-marrow stromal-cell dysfunction. Across these reviews, biological breadth is accompanied by the same translational requirement: standardized preparations, defined active constituents, and adequately powered clinical trials are needed before mechanistic promise can establish efficacy.

The systemic reach of metabolic disease is equally visible outside the traditionally emphasized liver, pancreas, and vasculature. Ayebazibwe et al. systematically reviewed 35 preclinical studies of hypoglycemic phytochemicals in diabetes-associated male reproductive dysfunction. The overall evidence linked improved glycemic control with lower oxidative stress, better sperm parameters and spermatogenesis, improved testicular morphology, and changes in testosterone, FSH, and LH; however, the evidence is exclusively preclinical and heterogeneous, making standardization and safety evaluation prerequisites for clinical translation. In high-risk pregnancies, da Fonseca et al. examine unsaturated fatty acids, particularly the omega-3 fatty acids EPA and DHA, as immunometabolic modulators. Their review highlights their effects on membrane composition, inflammatory signaling, inflammasome activation, mitochondrial function, and the biosynthesis of specialized pro-resolving mediators. Clinical evidence supports a potential role for omega-3 supplementation in reducing the risk of preterm birth, particularly among women with low baseline DHA status, whereas findings regarding preeclampsia and gestational diabetes remain heterogeneous. These observations highlight the potential value of precision nutrition strategies based on baseline nutritional assessment and biomarker-guided stratification. The systemic nature of metabolic regulation is also evident in hyperuricemia. Jin et al. demonstrate inter-organ coordination through a purified Paeonia lactiflora root fraction that lowered serum uric acid in a rat model by simultaneously inhibiting hepatic xanthine oxidase and adenosine deaminase and modulating renal transporters involved in urate reabsorption and secretion.

## Therapeutic evidence and clinical translation

The Research Topic also illustrates the wide evidentiary spectrum behind candidate metabolic therapies. Gul et al. studied troxerutin in high-fat-diet/streptozotocin diabetic male rats and reported dose-dependent improvements in lipid, hepatic, renal, and oxidative-stress measures, accompanied by upregulation of PPARalpha/PPARgamma and downregulation of FAS, SREBP-1c, TNF-alpha, and IL-6 across insulin-responsive tissues. These findings support a plausible metabolic and inflammatory mechanism, but they remain preclinical and should not be interpreted as evidence of equivalence to established human therapy. More broadly, the studies of troxerutin, Artemisia scoparia, Paeonia lactiflora, paeoniflorin, poliumoside, and botanical interventions in osteoporosis highlight both the pharmacological richness of natural products and the recurring challenges of chemical standardization, reproducibility, exposure, safety, and clinical confirmation.

Several papers move closer to direct clinical evidence. Zheng et al. combined a 6-month randomized, double-blind, placebo-controlled trial with network pharmacology, transcriptomics, machine learning, and a nested serum-proteomics analysis. Among 136 randomized participants with type 2 diabetes, 128 completed the study; Daixie Decoction granules added to metformin produced a modest HbA1c reduction versus placebo (−0.32%, P = 0.032). Computational and proteomic integration prioritized eight candidate targets (P2RX7, IL1B, PTPN1, AKT2, CD38, NFE2L2, NOS3, and MERTK), but these associations appropriately generate mechanistic hypotheses rather than prove causal target mediation. Jiang et al. synthesize 17 randomized trials including 1,549 participants and report that Huanglian Wendan Decoction added to conventional therapy improved multiple components of metabolic syndrome, including anthropometric, blood-pressure, glycemic, lipid, and insulin-resistance measures. The pooled estimates are encouraging, while certainty still depends on the methodological quality and heterogeneity of the underlying trials.

Two additional clinical contributions clarify that therapeutic value and mechanistic attribution should be separated. In a 12-week double-blind randomized trial of 30 women with elevated HOMA-IR receiving metformin, Ratajczak et al. found that a multistrain probiotic did not improve metabolic or anthropometric outcomes but significantly reduced gastrointestinal adverse symptoms. The finding expands the meaning of treatment benefit to tolerability and potential adherence rather than biomarker reduction alone. In a cross-sectional study of 185 participants with type 2 diabetes, Ismail et al., found that physical activity was independently associated with lower HbA1c, fasting glucose, HOMA-IR, and fructosamine, whereas circulating IL-6 was not independently associated with these metabolic outcomes and formal IL-6-by-activity interaction testing was negative. The study therefore supports a clinical association with physical activity while cautioning against assigning the observed benefit to a specific IL-6 mechanism based on cross-sectional data.

Taken together, these contributions illustrate the different levels of evidence involved in the development of new therapeutic strategies for metabolic diseases. Computational and multi-omics approaches can help identify candidate mechanisms, targets, and compounds, whereas experimental models provide an essential step for evaluating their biological relevance. Clinical studies are ultimately required to determine whether these findings translate into safe and effective therapeutic interventions. The studies included in this Research Topic therefore highlight the importance of integrating mechanistic discovery with progressive experimental and clinical validation.

## Conclusion

The 23 contributions included in this Research Topic reflect a broader view of metabolic disease, in which clinical and metabolic abnormalities arise from interconnected molecular, cellular, and systemic alterations. Although conventional metabolic parameters remain essential for diagnosis and therapeutic monitoring, they provide only a partial view of the underlying biological alterations. Future therapeutic progress will depend on identifying the mechanisms that are most relevant in each disease context, determining whether they can be effectively targeted, and validating these approaches across experimental and clinical settings. The studies presented in this Research Topic contribute to this effort and highlight the importance of strengthening the connection between mechanistic research and clinical translation.

